# Proteolysis-Targeting Chimera (PROTAC) Delivery into the Brain across the Blood-Brain Barrier

**DOI:** 10.3390/antib12030043

**Published:** 2023-06-26

**Authors:** Toshihiko Tashima

**Affiliations:** Tashima Laboratories of Arts and Sciences, 1239-5 Toriyama-cho, Kohoku-ku, Yokohama 222-0035, Japan; tashima_lab@yahoo.co.jp

**Keywords:** drug delivery into the brain, the BBB, receptor-mediated transcytosis, carrier-mediated transport, PROTAC, ubiquitin proteasome system, PROTAC-antibody conjugate, NanoPROTAC, Alzheimer’s disease, tau protein degradation

## Abstract

Drug development for neurodegenerative diseases such as Alzheimer’s disease, Parkinson’s disease, and Huntington’s disease has challenging difficulties due to the pharmacokinetic impermeability based on the blood-brain barrier (BBB) as well as the blurriness of pharmacodynamic targets based on their unclarified pathogenesis and complicated progression mechanisms. Thus, in order to produce innovative central nervous system (CNS) agents for patients suffering from CNS diseases, effective, selective delivery of CNS agents into the brain across the BBB should be developed. Currently, proteolysis-targeting chimeras (PROTACs) attract rising attention as a new modality to degrade arbitrary intracellular proteins by the ubiquitin-proteasome system. The internalizations of peptide-based PROTACs by cell-penetrating peptides and that of small molecule-based PROTACs through passive diffusion lack cell selectivity. Therefore, these approaches may bring off-target side effects due to wrong distribution. Furthermore, efflux transporters such as multiple drug resistance 1 (MDR1) expressed at the BBB might interrupt the entry of small molecule-based PROTACs into the brain. Nonetheless, intelligent delivery using machinery systems to absorb the nutrition into the brain for homeostasis, such as carrier-mediated transport (CMT) or receptor-mediated transcytosis (RMT), can be established. PROTACs with *N*-containing groups that are recognized by the proton-coupled organic cation antiporter might cross the BBB through CMT. PROTAC-antibody conjugates (PACs) might cross the BBB through RMT. Subsequently, such small molecule-based PROTACs released in the brain interstitial fluid would be transported into cells such as neurons through passive diffusion and then demonstrate arbitrary protein degradation. In this review, I introduce the potential and advantages of PROTAC delivery into the brain across the BBB through CMT or RMT using PACs in a non-invasive way.

## 1. Introduction

**Figure 1:**
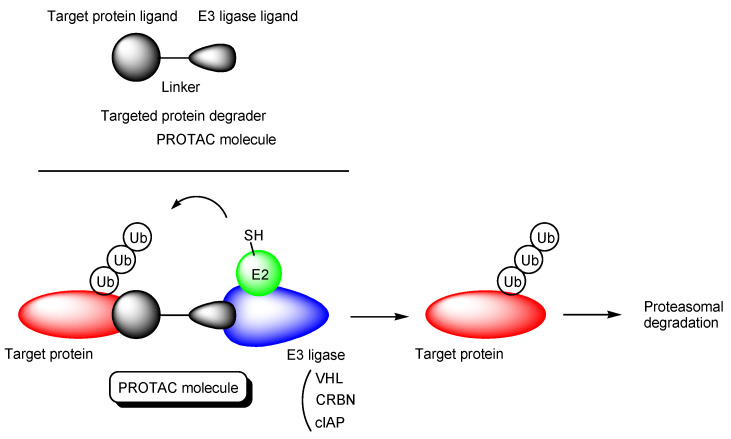
The structure of proteolysis-targeting chimeras (PROTACs) and the pathway of target protein degradation by PROTACs.

**Figure 2:**
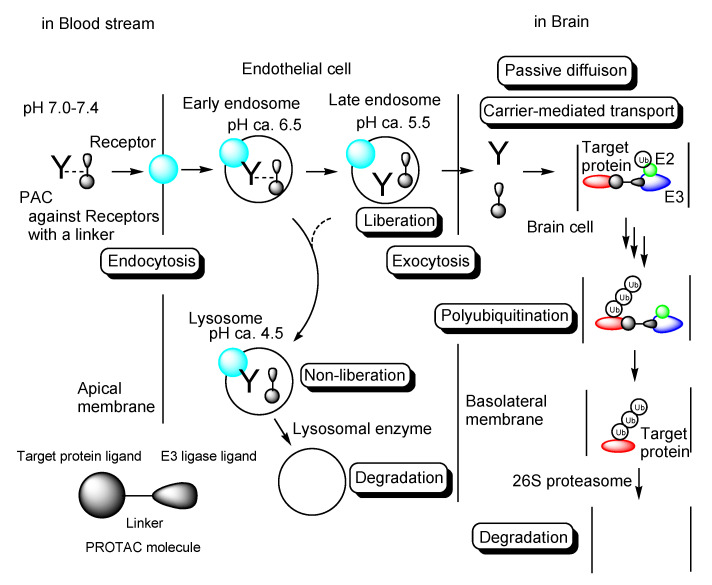
The pathway of intravenously administered PROTAC-antibody conjugates (PACs) against receptors (the light blue sphere) such as a transferrin receptor via a linker. PACs were internalized into the capillary endothelial cells.

It is true that medical treatment has benefited humankind in terms of health and longevity but the development of new pharmaceutical agents has become more and more difficult due to lack of seeds such as druggable targets and potent compounds, huge cost, adverse side-effects, and technological problems such as poor stability, poor solubility, cell membrane impermeability, and wrong distribution. There remain many intractable diseases that distress patients extremely. Thus, novel therapeutic modalities are required to resolve this present situation. Among them, the proteolysis-targeting chimera (PROTAC) (Figure 1) [1,2] is of current interest, because PROTAC molecules degrade arbitrary intracellular proteins by the ubiquitin-proteasome system. This chemical protein knockdown mechanism of the drug action is different from the mechanisms of inhibitors and antagonists, even though they are peptide-based or small molecule-based types. However, PROTACs have problems such as membrane permeability and cell selectivity. In drug discovery and development, cell membrane impermeability is a serious problem. Central nervous system (CNS) disease drugs must cross the blood-brain barrier (BBB) in order to be distributed into the brain. The BBB [3] is substantially composed of physical barriers such as (i) hydrophobic lipid bilayer membranes of the capillary endothelial cells, (ii) tight junctions between the capillary endothelial cells, (iii) periendothelial accessory backing structures formed by pericytes and astrocytes [4], and biological barriers such as (iv) excretion by efflux transporters including multiple drug resistance 1 (MDR1, P-glycoprotein) that captures hydrophobic low molecular substances just passing through the apical membrane of the capillary endothelial cells [5] and excretes them to the blood stream. Nonetheless, the brain must take in nutrients delivered by the blood and excrete waste products to the bloodstream to maintain physiological homeostasis. Glucose, amino acids, and insulin are transported at the BBB by corresponding solute carrier (SLC) transporters that constitute the superfamily. Moreover, high-molecular compounds are transported through receptor-mediated transcytosis or macropinocytosis. Generally, substances are categorized in size into low-molecular compounds (molecular weight (MW) < approximately 500 Da), middle-molecular compounds (MW approximately 500–3000 Da), and high-molecular compounds (MW > approximately 3000 Da). If designed low-molecular hydrophobic or hydrophilic compounds are the substrates of arbitrary SLC transporters at the BBB, they will be transported into the brain across the membrane of the capillary endothelial cells without being excreted by MDR1s. On the other hand, designed high- or middle-molecular compounds such as antibody-drug conjugates (ADCs) [6,7] or drug-encapsulated nanoparticles covered with antibodies would cross the BBB into the brain through the physical and biological systems such as receptor-mediated transcytosis or macropinocytosis based on the structuralism propounded by Dr. Claude Lévi-Strauss. So far, I have introduced the drug delivery systems across the membrane [6,7,8,9], using vectors such as antibodies [6,7,9], cell-penetrating peptides (CPPs) [9], tumor-homing peptides (THPs) [9], and transporter recognition units [8,9] that enable cell internalization. Designed compounds will behave as regulated by the physical and biological machinery systems. Large compounds cannot penetrate through the narrow pores of transporters and ion channels. Hydrophilic compounds cannot cross the lipidic membrane through passive diffusion. Positively charged substances will electrostatically interact with negatively charged substances. The ligand-receptor bindings will induce some types of biological events such as endocytosis and signal transduction. Substances never go against the rules of nature even at the molecular level, although human might recognize their behavior through statistical analyses in some cases. Hidden variable theory might elucidate such statistical behavior. Thus, well-designed compounds can be delivered into the brain across the BBB by taking advantage of such structural systems. In this perspective review, I introduce the possibilities and implements of PROTAC delivery into the brain across the BBB in a non-invasive way. A particularly promising strategy is PROTAC-antibody conjugates (PACs) through receptor-mediated transcytosis (Figure 2).

## 2. Discussion

### 2.1. PROTACs

**Figure 3:**
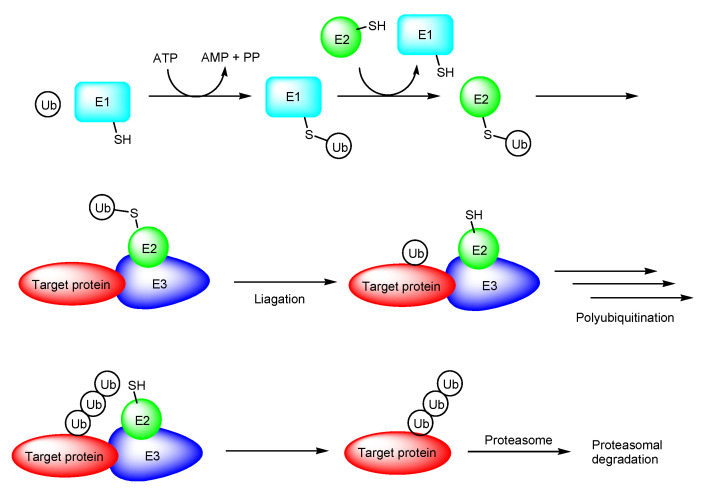
The pathway of ubiquitin proteasome system. Abbreviations: E1, ubiquitin-activating enzyme; E2, ubiquitin conjugase; E3, ubiquitin ligase; Ub, ubiquitin; PP, diphosphoric acid.

PROTAC is a novel therapeutic modality using the ubiquitin-proteasome system. PROTAC molecules degrade target proteins due to the endogenous decomposition system, different from the competitive inhibition or irreversible inhibition systems and slightly similar to the messenger RNA (mRNA) degradation system by short interfering RNAs (siRNA) called RNA interference. The ubiquitin-proteasome system is a crucial protein degradation system in all eukaryotes. Ubiquitin-activating enzymes (E1), ubiquitin conjugases (E2), ubiquitin ligases (E3), ubiquitin, and the 26S proteasome play essential roles in intracellular proteolysis (Figure 3) [10,11].

Canonical PROTACs are heterobifunctional molecules composed of a target protein ligand and an E3 ligase ligand via a suitable linker (Figure 1) [12]. They reversibly bind E3 ligases and target proteins also named proteins of interest (POIs). When they bind E3 ligases and target proteins simultaneously, polyubiquitination of target proteins occurs, which leads to the degradation by the 26S proteasomes. As an E3 ligase, VHL (von Hippel-Lindau), CRBN (cereblon), and cIAP (cellular inhibitor of apoptosis protein) are often targeted, although there are approximately 600 types of E3 ligases [13]. In many cases, linkers are polyethylene glycols (PEGs) and linear alkyl chains [14].

The permeability of small molecule-based PROTACs was evaluated in in vitro assay using cells. VHL-based PROTACs (*n* = 115) were classified into a high-permeability class (*n* = 46), a moderate-permeability class (*n* = 44), and a low-permeability class (*n* = 25). CRBN-based PROTACs (*n* = 113) were classified into a high-permeability class (*n* = 59), a moderate-permeability class (*n* = 45), and a low-permeability class (*n* = 9). Surprisingly, it turned out that small molecule-based PROTACs showed high and moderate membrane permeability. However, it was uncertain whether the transport mechanisms were based on passive diffusion or carrier-mediated transportation [15]. The stable solution conformations of PROTACs were determined by nuclear magnetic resonance (NMR) spectroscopy through nuclear Overhauser effect spectroscopy (NOESY) or molecular dynamics simulations. While the gauche effect of PEG-type linkers evoked a larger proportion of folded conformations, alkyl linkers evoked a larger proportion of elongated anti-conformations. It was likely that a PROTAC with PEG-type linkers that evoked folded conformation would be transported through passive diffusion to a greater extent than a PROTAC with alkyl linkers that evoked elongated anti-conformation in in vitro permeation assay calculating passive permeability using Caco-2 cell monolayers. Nevertheless, PROTACs with PEG-type linkers and PROTACs with alkyl linkers demonstrated permeation through the artificial membrane, to the same degree, in a parallel artificial membrane permeability assay (PAMPA) [16]. The prediction of the permeability will be improved by machine learning methods through physical property factors such as molecular weight, hetero atoms, H-bond donors, H-bond acceptors, the number of rotatable bonds (NRotB), logD, and the polar surface area (PSA).

**Figure 4:**
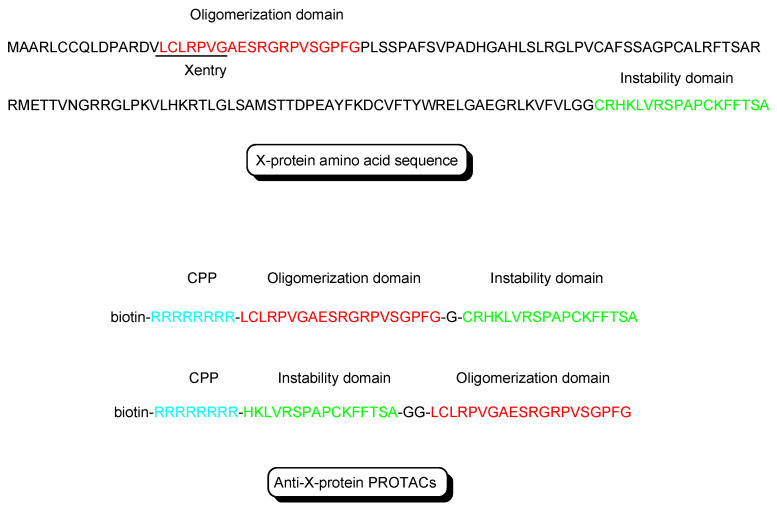
The structures of X-protein and anti-X-protein PROTACs. The oligomerization domain (shown in red) acts as an X-protein ligand. The instability domain (shown in green) as a degron peptide acts as an E3 ligase ligand. Although R8 is a main cell-penetrating peptide (CPP) (shown in blue) in the molecule, X-entry is a type of CPP.

**Figure 5:**
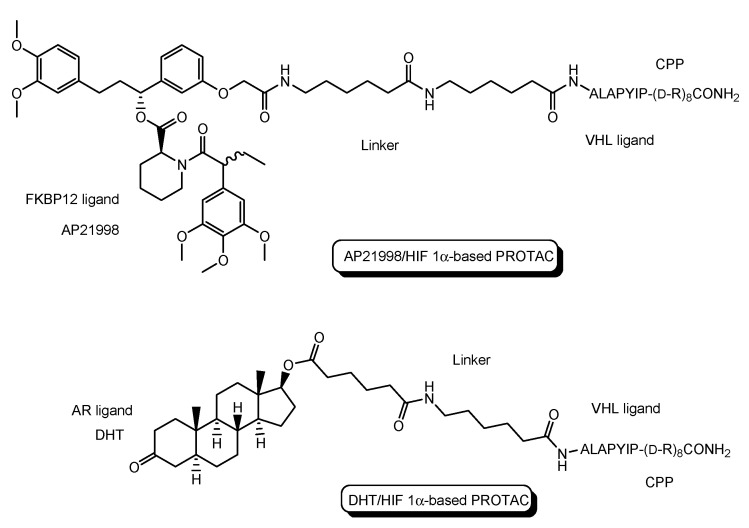
The structure of AP21998/ hypoxia inducible factor (HIF) 1α-based PROTAC, composed of AP21998 as a FK506 binding protein (FKBP12) ligand, the ALAPYIP sequence as a VHL ligand, and D-R8 as a cell-penetrating peptide (CPP), and dihydrotestosterone (DHT)/HIF 1α-based PROTAC, composed of DHT as an androgen receptor (AR) ligand, the ALAPYIP sequence as a VHL ligand, and D-R8.

**Figure 6:**
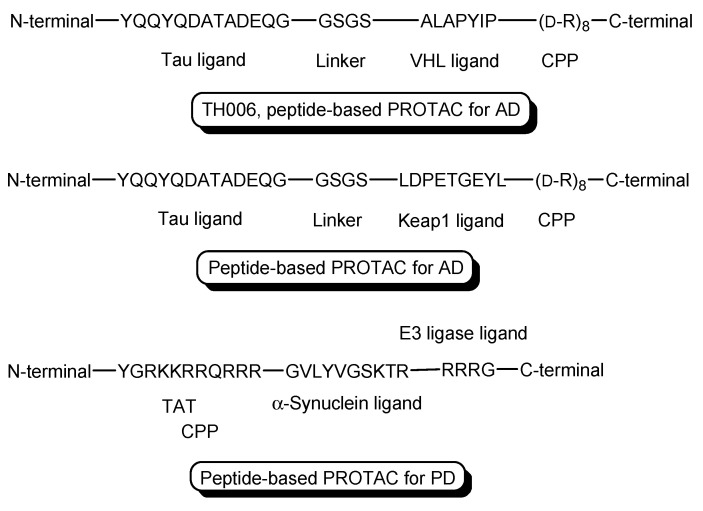
The structures of TH006 with a VHL ligand, a tau ligand, and D-R8 as a cell-penetrating peptide (CPP), a peptide-based PROTAC with Kelch-like ECH-associated protein-1 (Keap1) ligand as an E3 ligase ligand, a tau ligand, and D-R8, and a peptide-based PROTAC with an α-synuclein ligand, an E3 ligase ligand, and TAT as a CPP.

PROTACs, particularly peptide-based PROTACs, show poor permeability. To enhance their permeability, CPPs are introduced into PROTACs. CPPs are oligopeptides that are 5–30 residues in length and positively charged, and can enter cells across the membrane. Although the internalization mechanisms of CPPs remain unknown, receptor-mediated endocytosis and direct translocation are widely accepted. Trans-activator of transcription (TAT) protein (YGRKKRRQRRR), R9 (RRRRRRRRR), and penetratin (RQIKIWFQNRRMKWKK) are representative CPPs [9]. CPP-cargo conjugates can be used for drug delivery into cells across the membrane. Actually, PROTACs with CPP can enter cells and elicit degradation activities there. Several PROTACs with CPPs as a vector are shown here to understand membrane-permeable PROTAC molecule designs. Illustrations (Figure 4, Figure 5 and Figure 6) are easier to understand than text.

(i) X-protein, derived from the hepatitis B virus (HBV), induces hepatocellular carcinoma (HCC). Thus, the elimination of X-protein by a PROTAC approach can prevent HCC. X-proteins were oligomerized through the oligomerization domain that could be an X-protein ligand. Anti-X-protein peptide-based PROTACs with (a) the oligomerization domain of the X-protein as an X-protein ligand, (b) degron peptide of the X-protein as an E3 ligase ligand, and (c) R8 as a CPP (Figure 4) entered cells and destroyed the X-protein in HepG2 cells. In general, a degron is a peptide sequence within a protein and is recognized by an E3 ligase. The instability domain as a degron peptide would induce proteasomal degradation of the X-protein [17].
(ii) The first in vivo examples of small molecule-based PROTACs were demonstrated targeting the FK506 binding protein (FKBP12) or androgen receptor (AR). AP21998/hypoxia inducible factor (HIF) 1α-based PROTAC (Figure 5) is composed of (a) AP21998 as an FKBP12 ligand, (b) the ALAPYIP sequence as a VHL ligand, and (c) D-R8 fused to the *C*-terminus. FKBP12 was fused to enhanced green fluorescent protein (EGFP) by a vector, to monitor loss of intracellular fluorescence by degradation due to PROTACs. After hydroxylation of the central proline in the ALAPYIP sequence of HIF 1α by a proline hydroxylase, the hydroxylated ALAPYIP became recognized by VHL. On the other hand, dihydrotestosterone (DHT)/HIF 1α-based PROTAC (Figure 5) is composed of (a) DHT as an androgen receptor (AR) ligand, (b) the ALAPYIP sequence as a VHL ligand after hydroxylation of the central proline in the ALAPYIP sequence by a proline hydroxylase, and (c) D-R8 fused to the *C*-terminus. The AR was fused to GFP. In fact, the AP21998/ HIF 1α-based PROTAC degraded EGFP-FKBP12 in a VHL-dependent manner using HeLa^EGFP-FKBP^ cells. The fluorescence of FKBP12-EGFP was lost in the cells. The DHT/HIF1 α-based PROTAC induced the degradation of the AR using HEK293^GFP-AR^ cells [18]. These findings suggested that PROTACs were transported into cells across the membrane by virtue of CPPs. D-R8 is more stable to L-protein-mediated enzymatic degradation than L-R8.
(iii) PROTACs for Alzheimer’s disease (AD) were developed. The tau protein abnormally aggregates in AD patients’ brains. Peptide-based PROTACs with (a) a tau ligand, (b) an E3 ligase ligand, and (c) D-R8 fused to the *C*-terminus of the E3 ligase ligand were designed. Among them, TH006, with (b) the ALAPYIP sequence as a VHL ligand (Figure 6), demonstrated the highest tau degradation in in vitro assay using tau-overexpressed SH-SY5Y cells, and furthermore, it reduced the toxicity of amyloid β (Aβ), and lowered the tau level in an AD mouse model [19].
(iv) Moreover, peptide-based PROTACs with (a) a tau ligand, (b) Kelch-like ECH-associated protein-1 (Keap1) ligand as an E3 ligase ligand forming the Keap1- Cullin (Cul3) E3 ligase complex, and (c) D-R8 at the *C*-terminus (Figure 6) showed degradation of intracellular tau in vitro using tau-EGFP over-expressed SH-SY5Y cells [20].
(v) PROTACs for Parkinson’s disease (PD) were developed. α-Synuclein protein aggregation is a prominent feature in PD patients’ brains. Peptide-based PROTACs with (a) an α-synuclein ligand, (b) an E3 ligase ligand at the carboxyl terminus, and (c) TAT at the *N*-terminus (Figure 6) suppressed the cellular α-synuclein level in the primary cultured cortical neurons [21]. Although CPPs delivered PROTACs into cells, they lacked cell selectivity. Positively charged TAT was internalized through receptor-mediated endocytosis using ubiquitously expressed negatively charged heparan sulfate proteoglycans (HSPGs) as a receptor on the cell surface. Furthermore, passive diffusion also lacks cell selectivity. VHL and CRBN are ubiquitously expressed in various tissues [22]. To avoid wrong distribution and off-target side effects, cell-selective internalization should be established using well-designed compounds.

### 2.2. Clinical Trials of PROTACs

**Figure 7:**
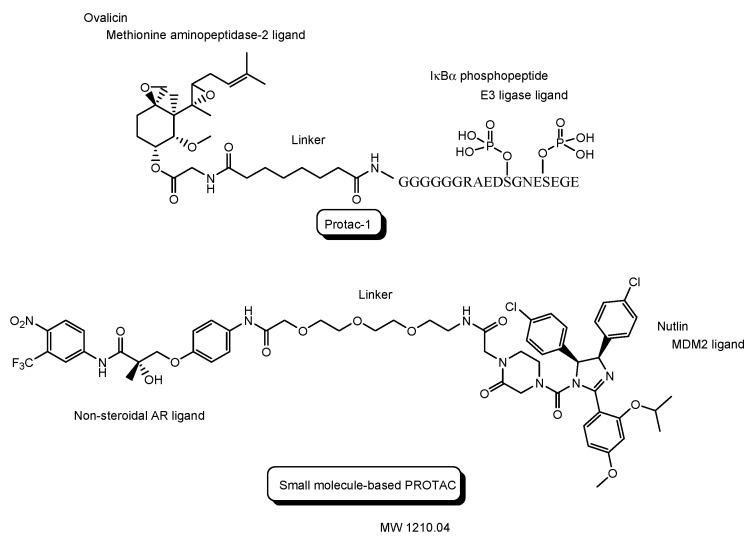
The structures of the first proteolysis-targeting chimera (PROTAC) and the first small molecule-based PROTAC.

**Figure 8:**
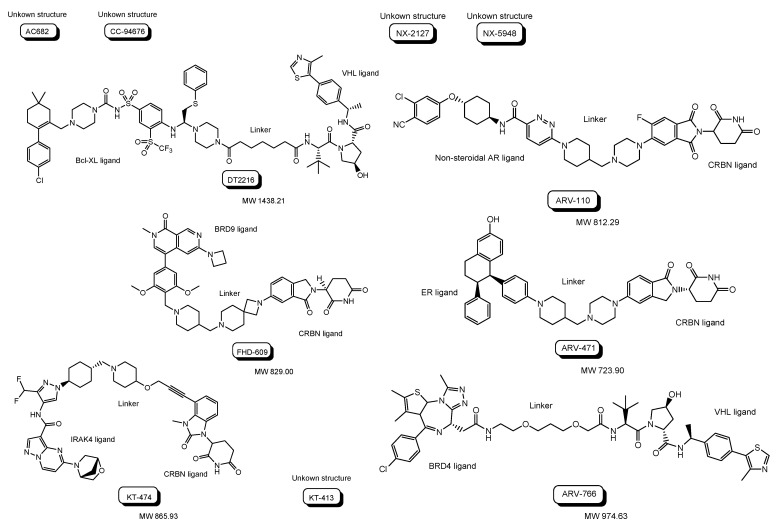
The structures of proteolysis-targeting chimeras (PROTACs) under clinical trials.

The first PROTAC, that is, Protac-1 (Figure 7), was reported in 2001. Protac-1 possesses ovalicin to bind methionine aminopeptidase-2 (MetAP-2) as a target protein ligand and the IκBα phosphopeptide to bind β-TRCP contained in the heterotetrameric Skp1-Cullin-F box E3 ligase complex as an E3 ligase ligand via an alkyl linker [23]. The first small molecule-based PROTAC with a non-steroidal AR ligand as a target protein ligand and nutlin as an E3 ligase ligand via a PEG-based linker (Figure 7), reported in 2008, effectively degraded AR after cell membrane permeation in cancer cells [24]. ARV-110 (Figure 8), with a non-steroidal AR ligand and a CRBN ligand, was the first PROTAC molecule that entered clinical testing in 2019, for the treatment of prostate cancer. A number of PROTACs have been developed. Clinical trials using PROTACs have been performed only for the treatment of cancers but, however, have not been performed for CNS diseases yet [25,26]. In general, the difficulty of CNS drug development is due to the impermeability of the BBB and wrong distribution. Thus, some improvements are required for PROTAC design. The PAC strategy can hopefully be one of the solutions.

Clinical trials using PROTACs for cancers (Table 1 and Figure 8) have been overlooked in order to horizontally deploy knowledge toward PROTACs for CNS diseases. Most of them are small or middle molecule-based PROTACs, probably due to manufacturability, stability, and membrane permeability through passive diffusion, although some of their structures are unfamiliar. The following are in phase 1 clinical trial: (i) AC682 with a CRBN ligand and an estrogen receptor (ER) ligand for breast cancer (NCT05080842), (ii) CC-94676 with a CRBN ligand and a non-steroidal AR ligand for prostatic neoplasms (NCT04428788), (iii) DT2216 with a VHL ligand and a B-cell lymphoma-extra large (Bcl-XL) ligand for solid tumor and hematologic malignancy (NCT04886622), (iv) FHD-609 with a CRBN ligand and a bromodomain-containing protein 9 (BRD9) ligand for advanced synovial sarcoma (NCT04965753), (v) KT-474 with a CRBN ligand and an interleukin 1 receptor associated kinase 4 (IRAK4) ligand for healthy volunteer atopic dermatitis, hidradenitis suppurativa (NCT04772885), (vi) KT-413 with a CRBN ligand and an IRAK4 ligand for non-Hodgkin lymphoma, diffuse large B cell lymphoma, DLBCL, and MYD88 gene mutation (NCT05233033), (vii) NX-2127 with a CRBN ligand and a Bruton tyrosine kinase (BTK) ligand for chronic lymphocytic leukemia (CLL), small lymphocyticlymphoma (SLL), Waldenstrom macroglobulinemia (WM), mantle cell lymphoma (MCL), marginal zone lymphoma (MZL), follicular lymphoma (FL), diffuse large B-cell lymphoma (DLBCL), primary CNS lymphoma (PCNSL) (NCT04830137), and (viii) NX-5948 with a CRBN ligand and a BTK ligand for CLL, SLL, DLBCL, FL, MCL, MZL, WM, and PCNSL (NCT05131022).

Furthermore, (ix) ARV-110 with a CRBN ligand and a non-steroidal AR ligand for prostate cancer metastatic (NCT03888612), (x) ARV-471 with a CRBN ligand and an ER ligand for breast cancer (NCT04072952), and (xi) ARV-766 with a VHL ligand and a BRD4 ligand for prostate cancer metastatic (NCT05067140), are in phase 1 and 2 clinical trials.

### 2.3. The Possibilities of PROTACs for CNS Diseases

CNS diseases remain unmet medical needs for patients suffering from neurodegenerative diseases such as AD, PD, and Huntington’s disease (HD) [27]. Innovative CNS pharmaceutical agents should be developed. PROTACs are promising candidates because of selective protein degradation, including Aβ and tau characteristics in the pathology of AD, α-synuclein characteristics in the pathology of PD, and huntingtin characteristics in the pathology of HD. Tau species cause tauopathies including AD, Pick’s disease (PiD), progressive supranuclear palsy (PSP), and corticobasal degeneration (CBD) [7]. Intravenously administered PROTAC molecules for CNS diseases must cross three plasma membranes, that is, the apical membrane and the basolateral membrane of the capillary endothelial cells at the BBB and the plasma membrane of target brain cells such as neurons, because the ubiquitin-proteasome system acts inside target brain cells. In general, canonical PROTAC molecules demonstrate low cell selectivity and low membrane permeability. Passive diffusion across the membrane lacks cell selectivity. HSPGs express ubiquitously on the surface of many types of cells. Thus, to avoid off-target side effects, high selective internalization into target cells, particularly, such as the capillary endothelial cells at the BBB should be carried out. (i) Receptor-mediated transcytosis using a transferrin receptor and an insulin receptor [6,7,9] and (ii) carrier-mediated transport using the proton-coupled organic cation antiporter [8] are non-invasively practicable as transendothelium strategies at the BBB. (i) A transferrin receptor is often used for receptor-mediated transcytosis. When a ligand in the bloodstream binds a transferrin receptor on the apical membrane of the capillary endothelial cells, ligand-receptor complexes are endocytosed. A ligand is liberated from the receptor in an endosome as acidification due to endosomal maturation and is subsequently exocytosed to the brain parenchyma through the brain interstitial fluid (ISF) based on the fusion between the endosome and the basolateral membrane via the secretory pathway instead of the degradation pathway leading to lysosomal degradation. Anti-transferrin receptor monoclonal antibodies are used as a ligand. Well-designed antibodies including antibody-PROTAC conjugates or PROTAC-encapsuled nanoparticles covered with antibodies can intentionally cross the BBB through receptor-mediated transcytosis. An IgG molecule often used for ADCs is approximately 14.2 nm in diameter and approximately 150 kDa. (ii) On the other hand, it is well-known that clinically used CNS drugs structurally possess *N*-containing groups. This fact suggests that arbitrary amine transporters at the BBB transport low molecular compounds with *N*-containing groups into the brain across the endothelium. The proton-coupled organic cation antiporter is considered as such an amine transporter, although its amino acid sequence and its topology are not identified yet [8]. Well-designed compounds with *N*-containing groups can intentionally cross the apical membrane into the capillary endothelial cells through carrier-mediated endocytosis. Therefore, well-designed PROTACs can cross the BBB through receptor-mediated transcytosis or through carrier-mediated endocytosis due to biologically and physically systemic structures based on the structuralism propounded by Dr. Lévi-Strauss.

### 2.4. The Implements of PROTACs for CNS Diseases

The ubiquitin-proteasome system can be used to treat AD [28]. In fact, tau was degraded by the ubiquitin-proteasome system during AD pathogenesis [29]. Interestingly, PROTACs for AD were evaluated. Whereas Aβ species were found in extracellular regions, tau species were found both in intracellular and extracellular regions [7]. PROTACs are suitable for tau clearance. Anti-tau peptide-based PROTACs with CPP (Figure 6) [19,20], including TH006, are described above. Anti-tau small molecule-based PROTACs are also being developed and are apt to be superior to peptide-based PROTACs with respect to manufacturability and stability.

**Figure 9:**
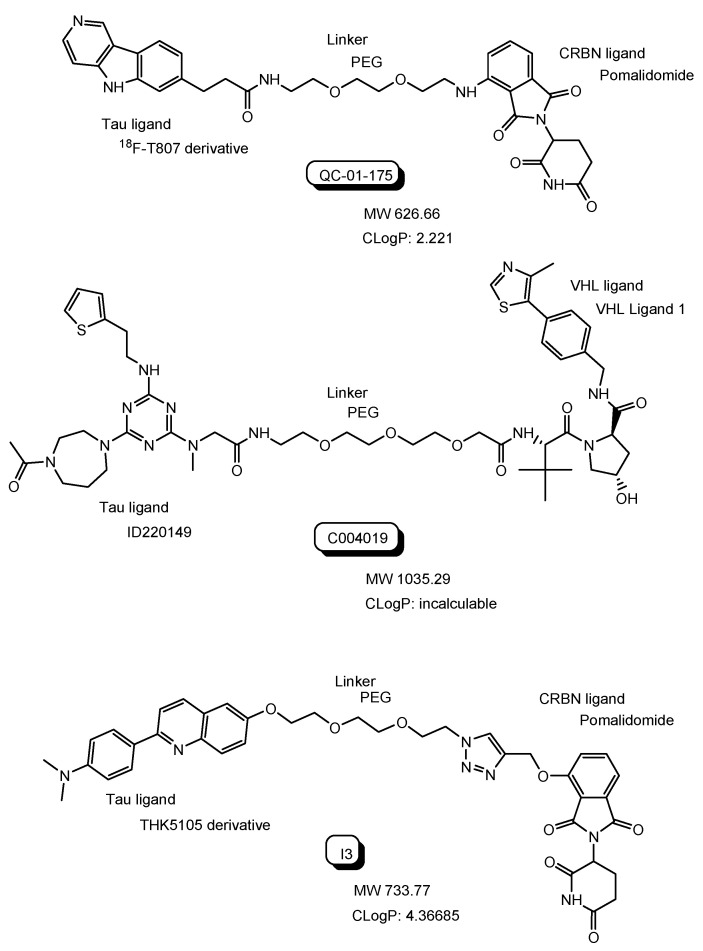
The structures of anti-tau PROTCs such as QC-01-175, C004019, and I3. The CLogP values were calculated by the software (ChemDraw Ultra version 7.0.1. provided from CambridgeSoft Corporation).

QC-01-175 with a tau ligand (^18^F-T807 derivative) and a CRBN ligand (Figure 9) degraded aberrant tau in frontotemporal dementia (FTD) patient-derived neuronal cell models in in vitro assay [30]. QC-01-175 was transported into the neuronal cell across the membrane, probably through passive diffusion due to the CLogP value of 2.221 or through carrier-mediated transportation. Lipinski’s rule of 5 proselytized by Dr. Christopher A. Lipinski suggested that oral drugs with good bioavailability should have a LogP value less than 5 and less than 500 MW [31]. Many PROTACs under clinical trials were perorally administered (Table 1), although their CLogP values were not calculated by the software and their MWs were beyond 500. Furthermore, it was revealed that small molecule-based PROTACs showed high and moderate membrane permeability in in vitro assay using cells [15,16]. Accordingly, it is likely that small molecule-based PROTACs will demonstrate decent membrane permeation through passive diffusion and/or through carrier-mediated transportation. It should be established whether small molecule-based PROTACs are substrates of MDR1 or not. Intriguingly, it was suggested that PROTAC-resistance in cancer cells resulted from MDR1 overexpression [32]. Therefore, approaches to avoid excretion by MDR1 are useful in PROTAC development. At the BBB, receptor-mediated transcytosis using a transferrin receptor and carrier-mediated endocytosis using the proton-coupled organic cation antiporter are solutions.

C004019 with a tau ligand (ID220149 [33]) and a VHL ligand (Figure 9) degraded tau proteins in the brain through subcutaneous administration in in vivo assay using wild-type, hTau-transgenic and 3xTg-AD mice with improvement of the synaptic and cognitive functions [34]. This finding implied that C004019 probably crossed the BBB through passive diffusion or through carrier-mediated transportation.

Excessive tau phosphorylation enhances the formation of neurofibrillary tangles (NFTs) containing tau and the neurotoxicity of Aβ. Anti-tau small molecule-based PROTACs with a tau ligand (THK5105 derivative) and a CRBN ligand, that is, compound I3, showed tau degradation and reduced Aβ-induced cytotoxicity in in vitro assay using the PC12 cells [35].

**Figure 10:**
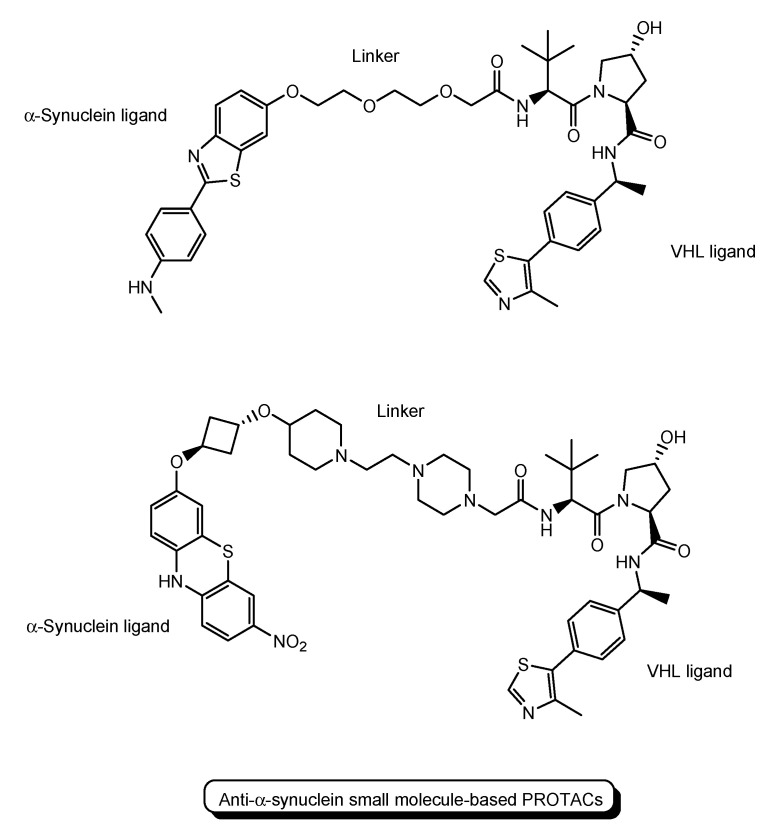
The structures of small molecule-based PROTACs for Parkinson’s disease (PD).

Furthermore, PROTACs for PD were also evaluated. A peptide-based PROTAC with an α-synuclein ligand, an E3 ligase ligand, and TAT demonstrated cellular α-synuclein reduction in the primary cultured cortical neuron, as mentioned above (Figure 6) [21]. Anti-α-synuclein small molecule-based PROTACs with a VHL ligand are also being developed (Figure 10) [36,37].

**Figure 11:**
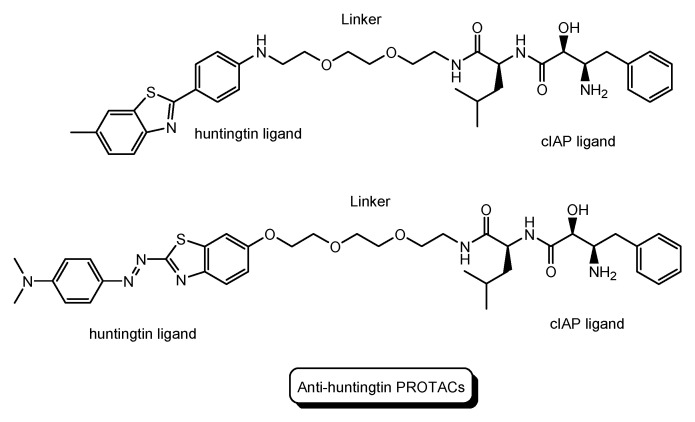
The structures of PROTACs for Huntington’s disease (HD).

HD is a neurodegenerative disease caused by heterogeneous aggregation of mutated huntingtin proteins with normal huntingtin proteins [38]. The pathogenesis of HD has still not been resolved [39]. Nonetheless, PROTACs for HD are being developed. Anti-huntingtin PROTAC with a huntingtin ligand and a cIAP ligand (Figure 11) reduced mutant huntingtin levels in fibroblasts derived from two patients with HD [40].

PROTACs can lower the intracellular tau and α-synuclein levels by degradation based on the ubiquitin-proteasome system. Nonetheless, how to deliver PROTACs selectively and effectively into the brain is an important problem. Carrier-mediated transport and receptor-mediated transcytosis are solutions.

### 2.5. Promising PROTACs for CNS Diseases

PROTACs for CNS diseases must enter the brain across the BBB. It was revealed that small molecule-based PROTACs showed high and moderate membrane permeability through passive diffusion [15,16]. Thus, they are distributed not only across the BBB to the brain but also to other tissues across certain membranes leading to off-target side effects. Some of them might be substrates of MDR1 at the BBB. Therefore, selective, effective transendothelium at the BBB should be conducted. It is known that transporters such as the proton-coupled organic cation antiporter [8] and receptors such as transferrin receptors and insulin receptors are expressed on the surface of the apical membrane of the capillary endothelial cells at the BBB. Two strategies for PROTACs to cross the BBB can be planned. One is carrier-mediated transport across the apical membrane using the proton-coupled organic cation antiporter and subsequent passive diffusion and/or carrier-mediated transport across the basolateral membrane, that is, the BBB. The other is receptor-mediated transcytosis across the BBB using a transferrin receptor. The BBB disruption caused by CNS diseases occurs and permits substances to enter spontaneously the brain through there [41]. However, this situation is transient and impermanent and depends on the conditions. Universal behaviors are necessary with respect to reproducibility.

Many existing small molecule-based PROTACs have *N*-containing groups. Thus, some of them might cross the BBB as substrates of the proton-coupled organic cation antiporter. If they are not the substrates, the introduction of *N*-containing groups such as the 2-(dimethylamino)ethyl group to PROTAC molecules might enable them to be the substrates. Prodrug approaches would be effective when the interaction of PROTACs to the corresponding intracellular receptors is interrupted due to steric hindrance based on *N*-containing groups. The permeability and degradation activity of designed PROTACs with *N*-containing groups can be tuned by repeated evaluation in in vitro tests reflecting the assay results.

Substance deliveries into the brain across the BBB were carried out through receptor-mediated transcytosis using a transferrin receptor. J-Brain Cargo^®^ is a drug delivery system into the brain across the BBB using anti-transferrin receptor ADCs with drugs [42]. Idursulfase beta [43], an anti-transferrin receptor antibody fused to iduronate-2-sulfatase based on J-Brain Cargo^®^, was clinically approved in Japan on 23 March 2021 for the treatment of Hunter syndrome. Anti-transferrin receptor ADCs can deliver drugs into the brain across the BBB through receptor-mediated transcytosis by the same strategy of J-Brain Cargo^®^. After the endocytosis triggered by ligand-receptor binding on the cell surface, antibodies are liberated from ADC-transferrin receptor complexes in endosomes as acidification. pH in endosomes gradually reduces from the early endosome (pH approximately 6.5) to late endosome (pH approximately 5.5) and then lysosome (pH approximately 4.5) by vacuolar H^+^-ATPase proton pumps [9]. pH-sensitive linkers connecting an antibody and drugs are cleaved in such acidification. Eventually, liberated antibodies and drugs are released to the brain based on the fusion between endosomes and the membrane in the secretory pathway. When the affinity between the antibody and a transferrin receptor is high, unliberated ADC-transferrin receptor complexes are degraded in lysosomes by lysosomal enzymes in the degradation pathway. The affinity between the antibody and a transferrin receptor should be moderate.

PROTAC-antibody conjugate (PAC) is a type of ADC, replacing ADC drugs with PROTACs. PACs, also called degrader-antibody conjugates (DACs), have attracted attention [44,45].

**Figure 12:**
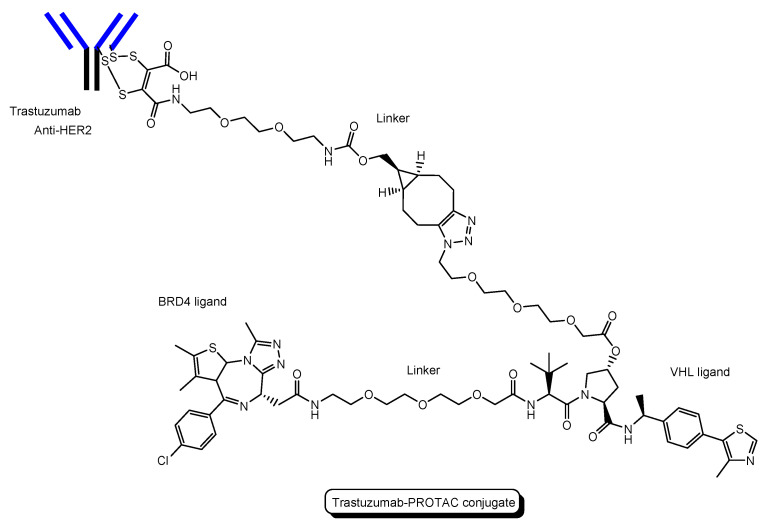
The structure of trastuzumab-PROTAC conjugate with a BRD4 ligand and a VHL ligand.

**Figure 13:**
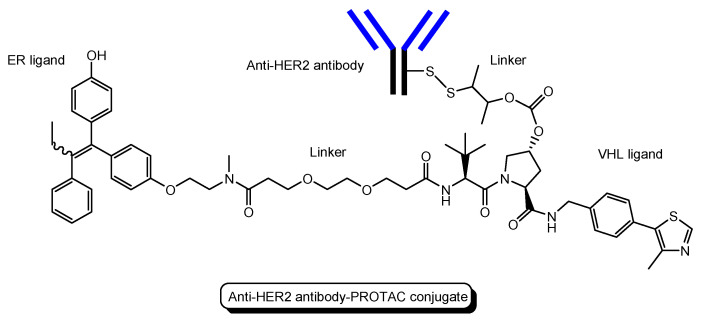
The structure of anti-HER2 antibody-PROTAC conjugate with an ER ligand and a VHL ligand.

**Figure 14:**
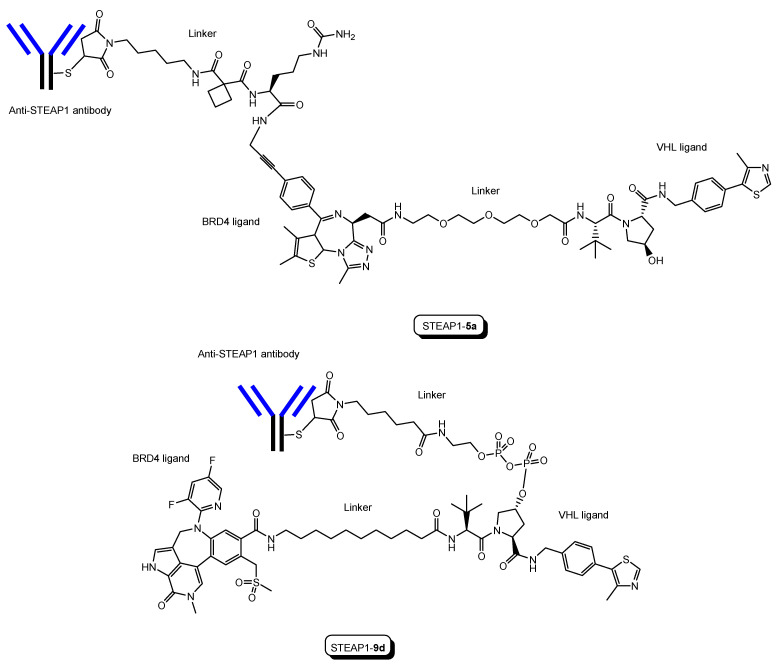
The structures of anti-STEAP1 antibody-PROTAC conjugates, STEAP1-**5a** and STEAP1-**9d**, with a BRD4 ligand and a VHL ligand.

(i) A trastuzumab-PROTAC conjugate with a BRD4 ligand and a VHL ligand (Figure 12) showed BRD4 degradation only in HER2 positive breast cancer cell lines [46]. Trastuzumab (Herceptin) is a humanized, recombinant monoclonal antibody against the extracellular domain of human epidermal growth factor type 2 (HER2). This trastuzumab-PROTAC was internalized into cells through receptor-mediated endocytosis using HER2. It was thought that S-S bonds that connected linkers and a trastuzumab were reductively cleaved in endosomes [47] and that ester bonds that connected a linker and a PROTAC were enzymatically cleaved in endosomes and/or lysosomes. Cleaved PROTACs were implied to have been transported into the cytosol across the membrane of endosomes and/or lysosomes through passive diffusion, because PROTACs showed BRD4 degradation by the ubiquitin-proteasome system.
(ii) Several antibody-PROTAC conjugates with an ER ligand and an E3 ligase ligand were designed and evaluated for their ER degradation activity. Among them, an anti-HER2 antibody-PROTAC conjugate with an ER ligand and a VHL ligand (Figure 13) demonstrated the highest ERα degradation (99%) using MCF7-neo/HER2 cells that were stably transfected to overexpress HER2 [48]. Thus, this PAC entered cells through receptor-mediated endocytosis using HER2. It was thought that cleaved PROTACs were transported into the cytosol across the membrane of endosomes and/or lysosomes through passive diffusion.
(iii) Six-transmembrane epithelial antigen of prostate 1 (STEAP1) is a membrane protein overexpressed in cancer cells. Anti-STEAP1 antibody-PROTAC conjugates with a BRD4 ligand and a VHL ligand, particularly STEAP1-**5a** (drug-to-antibody ratio (DAR) 6.0) (Figure 14), afforded degradation of the BRD4 protein with a DC_50_ value of 0.67 nM using PC3-S1 prostate cancer cells [49]. The DC_50_ value is the concentration at which the target is degraded by fifty percent. Modified anti-STEAP1 antibody-PROTAC conjugates with a BRD4 ligand and a VHL ligand, particularly STEAP1-**9d** (DAR 5.9) (Figure 12), afforded the highest degradation of the BRD4 protein with a DC_50_ value of 0.025 nM using PC3-S1 prostate cancer cells [50]. Anti-STEAP1 antibody-PROTAC conjugates entered cells through receptor-mediated endocytosis using STEAP1. Enzymatically cleaved PROTACs were transferred to the cytosol from lysosomes probably through passive diffusion. Whereas STEAP1-**5a** was cut by proteases, STEAP1-**9d** was cut by phosphatases. Currently, PACs have been investigated mainly for cancer therapy. However, this strategy can be applied to other diseases including CNS diseases.

Intravenously administered anti-transferrin receptor PACs would be delivered into the brain across the BBB, similarly to the anti-transferrin receptor ADC (Figure 1). If PACs are tethered with a pH-sensitive linker, liberated PROTACs in endosomes would be released into the brain and be transported into cells. Thus, anti-transferrin receptor PAC strategy is a promising method to deliver PROTACs into the brain across the BBB.

Moreover, PACs have a long half-life based on neonatal Fc receptor (FcRn)-mediated salvation from the lysosomal degradation. FcRn is expressed at the apical membrane of the endothelial cells. Under physiological pH (7.0–7.4), antibodies and FcRn do not bind in the systemic circulation. Endocytosed by-stander antibodies bind FcRn under weak acidic conditions in endosomes as acidification. Antibody-FcRn complexes in endosome were exposed to the systemic circulation through exocytosis in the secretory pathway. Antibody-FcRn complexes were liberated under physiological pH [6,7]. The half-lives of the antibodies were 29.7 days for IgG1, 26.9 days for IgG2, and 15.7 days for IgG3 [51]. Therefore, PACs are expected to have long half-life, resulting in low dose administration.

Nanoparticles as carriers used for drug delivery systems are biodegradable, biocompatible, stable, and easy to modify. NanoPROTACs are PROTACs using nanoparticles and make it possible to improve the delivery and mechanism of action of PROTACs [52]. PROTAC-encapsulated nanoparticles covered with antibodies were developed. PROTAC with a BRD4 ligand and a VHL ligand, that is, MZ1, was encapsulated in nanoparticles covered with trastuzumab targeting HER2. This NanoPROTAC (100 nm in diameter) showed a significant cytotoxic effect in in vitro assay using HER2-positive breast cancer cell lines [53]. Most endocytosis is (a) clathrin-dependent endocytosis (endosomal diameter of 85–150 nm), although there are several other mechanisms of endocytosis such as (b) caveolae-dependent endocytosis (endosomal diameter of 50–100 nm), (c) clathrin- and caveolae-independent endocytosis (endosomal diameter of approximately 90 nm), (d) macropinocytosis (endosomal diameter of 0.2–5 µm), and (e) other mechanically incomprehensible endocytoses [9]. The endocytosed substances must be of a size that can be contained in endosomes. After receptor-mediated endocytosis using HER2, MZ1 molecules were released through nanoparticles composed of poly-lactic acid (PLA) and showed the degradation via endosomal/lysosomal escape. Thus, similarly, PROTAC-encapsulated nanoparticles covered with anti-transferrin receptor antibodies might cross the BBB through receptor-mediated transcytosis. PROTACs with a tau ligand and an E3 ligase ligand might be released from the nanoparticles in endosomes and/or in the brain ISF and then penetrate neurons through passive diffusion. This NanoPROTAC strategy would be a promising method to deliver PROTACs into the brain across the BBB.

## 3. Conclusions

Neurodegenerative diseases such as AD, PD, and HD still remain intractable, because methods of effective treatment have not been established due to unclarified pathogenesis and complicated progression mechanisms. Moreover, most drugs do not reach the brain due to the impermeability based on the BBB. Innovative CNS agents to cross the BBB and elicit their activity there should be developed. At present, PROTAC is attention-getting as a novel modality. PROTAC molecules are constituted of heterobifunctional units of a target protein ligand and a E3 ligase ligand via a linker and induce the proteasomal degradation of the target proteins by the ubiquitin-proteasome system (Figure 1). They are structurally divided into peptide-based type and small molecule-based types. Peptide-based PROTACs probably show poor permeability and thus use CPPs such as R8 to cross the membrane. On the other hand, small molecule-based PROTACs show high and moderate permeability through passive diffusion [15,16]. The internalizations based on CPPs and passive diffusion are subject to off-target side effects due to wrong distribution. It is suggested that small molecule-based PROTACs are substrates of MDR1 that interrupt the entry into the brain. Therefore, selective distribution and effective internalization should be accomplished simultaneously by taking advantage of the biological and physical machinery systems based on structuralism. This is assimilation rather than hijack. The brain takes in nutrition from the systemic circulation across the BBB through carrier-mediated transport, receptor-mediated transcytosis, and macropinocytosis. For example: (i) it is known that drugs with *N*-containing groups are delivered into the brain across the BBB. Amine derivatives are positively charged in the systemic circulation under physiological conditions and cannot penetrate the membrane through passive diffusion. Amine transporters absorb drugs with *N*-containing groups. The proton-coupled organic cation antiporter is suggested to mediate the absorption of drugs with *N*-containing groups, although its amino acid sequence has not been identified yet. Transporter-consciously designed compounds with *N*-containing groups can be transported in the capillary endothelial at the BBB. (ii) Some receptors such as transferrin receptors and insulin receptors at the apical membrane in the capillary endothelial cells deliver substances into the brain through receptor-mediated transcytosis. In the case of transferrin receptors, holo-transferrin (apo-transferrin-Fe (III) complex) binding to transferrin receptors mediates endocytosis. Holo-transferrin is liberated from the complex receptor as endosomal acidification and subsequently is released to the brain ISF through exocytosis based on the fusion between endosomes and the basolateral membrane. Artificial ligands of transferrin receptors, such as antibodies, induce receptor-mediated transcytosis. (iii) Endosomes triggered by clathrin-dependent endocytosis are 85–150 nm in diameter, which is enough to contain nanoparticles. Drugs encapsulated in nanoparticles are protected from enzymatic metabolism, like RNAs in exosomes. Anti-receptor antibody-loaded nanoparticles are ligands to the corresponding receptors that mediate transcytosis at the BBB. The clustering of ligand-receptor complexes derived from transferrin receptors enhances endocytosis [54,55]. Anti-receptor antibody-loaded nanoparticles as a multiligand might enhance receptor-mediated transcytosis at the BBB.

Therefore, as a summary, the delivery methods of PROTACs into the brain across the BBB are presented as three approaches: (i) PROTACs with *N*-containing groups that are recognized by the proton-coupled organic cation antiporter [8], (ii) PROTAC-antibody conjugate (PAC) targeting transferrin receptors (Figure 2), and (iii) PROTAC-encapsulated nanoparticles covered with antibodies targeting transferrin receptors as a NanoPROTAC strategy.

Inspired readers are expected to suggest better ideas (Table 2). Substances materialistically and autonomously act in biological and physical machinery systems that regulate ATP-driven organic activities in tissues, cells, and organelles, based on the structuralism propounded by Dr. Lévi-Strauss [56,57]. Humans cannot intentionally control the pharmacokinetic behavior of such substances within a living organism, unless they use psychokinesis. However, comprehensive drug design incorporating the structuralism based on existentialism proselytized by Dr. Jean-Paul Sartre can overcome the restrictions of the present systemic structures to elicit their activity pharmacokinetically and pharmacodynamically. As a result, medicinal chemists and pharmaceutical scientists will produce innovative drugs that treat intractable diseases.

## Figures and Tables

**Table 1:** Summary of clinical trials focusing on proteolysis-targeting chimeras (PROTACs) described in this review.

#	Drug	Route	E3 Ligand	Target Protein Ligand	Disease	Sponsor	Phase	Study Start Date	Study Completion Date	ClinicalTrials.gov Identifier (accessed on 15 January 2023)	Status
(i)	AC682	Oral	CRBN	ER	Cancer	Accutar Biotechnology, Inc.	Phase 1	12 November 2021	September 2023	NCT05080842	Recruiting
(ii)	CC-94676	Oral	CRBN	Non-steroidal AR	Cancer	Celgene	Phase 1	22 June 2020	27 February 2025	NCT04428788	Recruiting
(iii)	DT2216	Intravenous	VHL	Bcl-XL	Cancer	Dialectic Therapeutics, Inc.	Phase 1	25 August 2021	15 April 2023	NCT04886622	Recruiting
(iv)	FHD-609	Intravenous	CRBN	BRD9	Cancer	Foghorn Therapeutics, Inc.	Phase 1	17 August 2021	31 May 2025	NCT04965753	Recruiting
(v)	KT-474	Oral	CRBN	IRAK4	Cancer	Kymera Therapeutics, Inc.	Phase 1	23 February 2021	20 October 2022	NCT04772885	Completed
(vi)	KT-413	Intravenous	CRBN	IRAK4	Cancer	Kymera Therapeutics, Inc.	Phase 1	13 June 2022	May 2025	NCT05233033	Recruiting
(vii)	NX-2127	Oral	CRBN	BTK	Cancer	Nurix Therapeutics, Inc.	Phase 1	5 May 2021	November 2023	NCT04830137	Recruiting
(viii)	NX-5948	Oral	CRBN	BTK	Cancer	Nurix Therapeutics, Inc.	Phase 1	13 April 2022	May 2024	NCT05131022	Recruiting
(ix)	ARV-110	Oral	CRBN	Non-steroidal AR	Cancer	Arvinas Androgen Receptor, Inc.	Phase 1 Phase 2	1 March 2019	31 October 2023	NCT03888612	Recruiting
(x)	ARV-471	Oral	CRBN	ER ligand	Cancer	Arvinas Estrogen Receptor, Inc.	Phase 1 Phase 2	5 August 2019	June 2023	NCT04072952	Recruiting
(xi)	ARV-766	Oral	VHL	BRD4	Cancer	Arvinas Androgen Receptor, Inc.	Phase 1 Phase 2	2 September 2021	27 June 2025	NCT05067140	Recruiting

**Table 2:** All compounds introduced in this perspective review.

#	Administrated drug	Formulation	Receptor for endocytosis	Disease	Vector	Target Protein Ligand	E3 Ligand	Status	References
(1)	Anti-X-protein peptide-based PROTACs	PROTACs with the oligomerization domain, degron peptide, and R8	Heparan sulfate proteoglycans	Cancer	R8 and X-entry as CPPs	Oligomerization domain of the X-protein as an X-protein ligand	Degron peptide of the X-protein as an E3 ligand	In vitro basic research	[17]
(2)	AP21998/ hypoxia inducible factor (HIF) 1α-based PROTAC	PROTAC with AP21998, ALAPYIP sequence, and D-R8	Heparan sulfate proteoglycans	-	D-R8 as a CPP	AP21998 as an FK506 binding protein (FKBP12) ligand,	ALAPYIP sequence as a VHL ligand	In vitro Basic research	[18]
(3)	Dihydrotestosterone (DHT)/HIF 1α-based PROTAC	PROTAC with DHT, ALAPYIP sequence, and D-R8	Heparan sulfate proteoglycans	Cancer	D-R as a CPP	DHT as an androgen receptor (AR) ligand	ALAPYIP sequence as a VHL ligand	In vitro Basic research	[18]
(4)	TH006	Peptide-based PROTAC with a tau ligand, ALAPYIP sequence, and D-R8	Heparan sulfate proteoglycans	Alzheimer’s disease	D-R as a CPP	Tau ligand	ALAPYIP sequence as a VHL ligand	In vitro Basic research	[19]
(5)	Peptide-based PROTAC	Peptide-based PROTAC with a tau ligand, Kelch-like ECH-associated protein-1 (Keap1) ligand, and D-R8	Heparan sulfate proteoglycans	Alzheimer’s disease	D-R as a CPP	Tau ligand	Keap1 ligand as an E3 ligase ligand	In vitro Basic research	[20]
(6)	Peptide-based PROTAC	Peptide-based PROTACs with an α-synuclein ligand, the E3 ligase ligand, and TAT	Heparan sulfate proteoglycans	Parkinson’s disease	TAT as a CPP	α-Synuclein ligand	E3 ligase ligand	In vitro Basic research	[21]
(7)	Protac-1 as the first PROTAC	Protac with ovalicin and the IκBα phosphopeptide	-	-	--	Ovalicin to bind methionine aminopeptidase-2	IκBα phosphopeptide to bind β-TRCP contained in the Skp1-Cullin-F box E3 ligase complex	In vitro Basic research	[23]
(8)	The first small molecule-based PROTAC	PROTAC with a non-steroidal AR ligand and nutlin	-	Cancer	-	Non-steroidal AR ligand	Nutlin as an E3 ligase ligand	In vitro Basic research	[24]
(9)	ARV-110 as the first PROTAC entering the clinical trial test	PTOTAC with a non-steroidal AR ligand and a CRBN ligand	-	Cancer	-	Non-steroidal AR ligand	CRBN ligand	phase 1 phase 2	(Table 1) [25,26]
(10)	Eight PROTACs under clinical trials	Small molecule-based PROTAC with a target protein ligand and an E3 ligase ligand	-	Canceer	-	Target protein ligand	E3 ligase ligand	phase 1	(Table 1) [25,26]
(11)	Three PROTACs under clinical trials including ARV-110	Small molecule-based PROTAC with a target protein ligand and an E3 ligase ligand	-	Cancer	-	Target protein ligand	E3 ligase ligand	phase 1 phase 2	(Table 1) [25,26]
(12)	QC-01-175	PROTAC with ^18^F-T807 derivative and a CRBN ligand	-	Alzheimer’s disease	-	^18^F-T807 derivative as a tau ligand	CRBN ligand	In vitro Basic research	[30]
(13)	C004019	PROTAC with ID220149 and a VHL ligand	-	Alzheimer’s disease	-	ID220149 as a tau ligand	VHL ligand	In vivo Basic research	[34]
(14)	I3	PROTACs with THK5105 derivative and a CRBN ligand	-	Alzheimer’s disease	-	THK5105 derivative as a tau ligand	CRBN ligand	In vitro Basic research	[35]
(15)	Small molecule-based PROTACs	PROTACs with an α-synuclein ligand and a VHL ligand	-	Parkinson’s disease	-	α-Synuclein ligand	VHL ligand	In vitro Basic research	[36,37]
(16)	Small molecule-based PROTACs	PROTACs with a huntingtin ligand and a cIAP ligand	-	Huntington’s disease	-	Huntingtin ligand	cIAP ligand	In vitro Basic research	[40]
(17)	Idursulfase beta	Anti-transferrin receptor antibody fused to iduronate-2-sulfatase	Transferrin receptor	Hunter syndrome	Anti-transferrin receptor antibody	-	-	Launched in 2021	[43]
(18)	Trastuzumab-PROTAC conjugate	PROTAC-antibody conjugate with a BRD4 ligand and a VHL ligand	HER2	Cancer	Anti-HER2 antibody	BRD4 ligand	VHL ligand	In vitro Basic research	[46]
(19)	Anti-HER2 antibody-PROTAC conjugate	PROTAC-antibody conjugate with an ER ligand and a VHL ligand	HER2	Cancer	Anti-HER2 antibody	ER ligand	VHL ligand	In vitro Basic research	[48]
(20)	STEAP1-**5a**	PROTAC-antibody conjugate with a BRD4 ligand and a VHL ligand	STEAP1	Cancer	Anti-STEAP1 antibody	BRD4 ligand	VHL ligand	In vitro Basic research	[49]
(21)	STEAP1-**9d**	PROTAC-antibody conjugate with a BRD4 ligand and a VHL ligand	STEAP1	Cancer	Anti-STEAP1 antibody	BRD4 ligand	VHL ligand	In vitro Basic research	[50]
(22)	NanoPROTACs covered with trastuzumab	PROTAC-encapsulated nanoparticles covered with antibodies	HER2	Cancer	Anti-HER2 antibody	BRD4 ligand	VHL ligand	In vitro Basic research	[53]
(23)	PROTAC-antibody conjugate	PROTAC-antibody conjugate with a tau ligand and an E3 ligase ligand	Transferrin receptor	Alzheimer’s disease	Anti-transferrin receptor antibody	Tau ligand	E3 ligase ligand	Under analysis in Tashima lab	-
(24)	NanoPROTACs covered with antibodies	PROTAC-encapsulated nanoparticles covered with antibodies	Transferrin receptor	Alzheimer’s disease	Anti-transferrin receptor antibody	Tau ligand	E3 ligase ligand	Under analysis in Tashima lab	-

## Data Availability

Not applicable.
